# Knowledge translation in the reality of Brazilian public health

**DOI:** 10.11606/s1518-8787.2020054002073

**Published:** 2020-07-20

**Authors:** Keitty Regina Cordeiro de Andrade, Maurício Gomes Pereira

**Affiliations:** I Universidade de Brasília Faculdade de Medicina Departamento de Medicina BrasíliaDF Brasil Universidade de Brasília. Faculdade de Medicina. Departamento de Medicina. Brasília, DF, Brasil

**Keywords:** Implementation Science, Information Dissemination, Translational Medical Research, Health Communication, Public Health

## Abstract

The term knowledge translation has been used to describe the process of applying research results to the real world, in order to enhance the quality and effectiveness of health care and services. The aim of this article is to discuss the incorporation of knowledge translation in the Brazilian public health. The article addresses the basic activities of knowledge translation and lists challenges and perspectives in Brazilian scenario. Brazil began to move towards understanding the process of translating scientific knowledge into practice. Investing in pilot studies to adapt the so-called effective interventions to the Brazilian scenario may be an alternative. Increasing the qualification of Brazilian researchers in the design and evaluation of implementation studies is relevant to improve this field in the country.

## INTRODUCTION

The application of real-life search results is an old challenge that persists to this day^1^. Traditionally, the implementation of new knowledge takes many years, which makes it difficult to innovate in the provision of health services, results in inefficiency of health systems and impacts on the population’s quality of life^2^. Considering this difficulty, efforts have emerged to ensure that evidence is effectively understood and implemented in health practices^3^.

There is no consensus among the terms used to describe these efforts^4^. Research utilization, implementation science, knowledge translation, knowledge transfer, and knowledge mobilization are expressions often employed^4-6^. Some also suggest the use of K* (knowledge star)^7^. In this article, we will use knowledge translation because it is the most used worldwide^8^.

Some middle- and low-income countries have advanced in understanding and executing strategies for knowledge translation^9^. What about Brazil? What are the obstacles and advances of KT? Are research results used to formulate guidelines for practices, policies and programs? Are the guidelines for practice and options for policies and programs implemented? These questions motivated the elaboration of this article, which discusses concepts that describe some of the complexities that influence knowledge translation in the Brazilian public health.

## WHAT IS KNOWLEDGE TRANSLATION?

Knowledge translation is one of several terms used to describe the science of putting evidence into action and understanding how evidence-based practices work in the real world. It is an interactive process of knowledge that includes the synthesis, dissemination, exchange and use of knowledge in order to improve services and make effective products available to the population and, thus, strengthen the health system. Table 1 shows the elements that compose a definition of knowledge translation and its respective descriptions^7,9^.

**Table 1:** Elements that make up the definition of knowledge translation and their description.

Elements of knowledge	Description
Synthesis	Contextualization and integration of the results of individual research on the subject.
Dissemination	Knowledge transfer through the identification of the target audience, personalization of the message and definition of the best means of communication.
Exchange	Interaction between knowledge producers and users, aiming at mutual learning by an active partnership to solve a certain problem.
Application	Use of knowledge in real life through activities consistent with ethical and cultural principles, as well as legal and regulatory structures.

Note: Adapted from Straus et al.^**5**^

Numerous models represent the components needed for knowledge translation^10^. The common aspects among them focus on the suggestion of overcoming the traditional ways of disseminating a new discovery^11^. The Figure shows a knowledge roundabout, analogous to a transit roundabout, where the continuous flow of traffic around the central island encompasses dynamic phases for the use of evidence. It represents the idea of knowledge in motion, which when synthesized and transferred is presumably better implemented by users.

**Figure:**
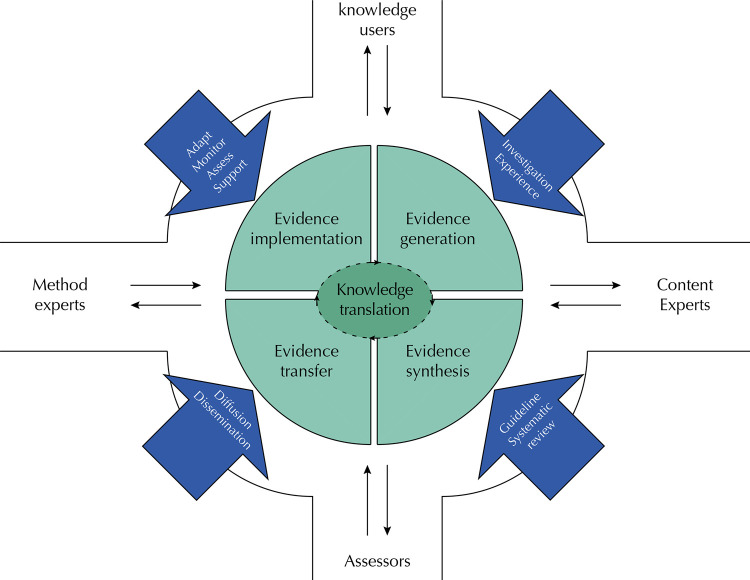
Knowledge translation roundabout. The continuous traffic flow around the central island represents activities for knowledge translation that can start at any stage of the process. Namely: *evidence generation, comprises the primary studies essential to support future research; evidence synthesis, compiles the results of primary studies to determine what is known about a problem and interpret them in the context of global evidence; evidence transfer, employs strategies for diffusion or dissemination of knowledge to the potential user; and evidence implementation, uses methods to adopt evidence-based interventions and understand how and why they work in certain contexts. Vehicles entering and leaving the roundabout represent the interaction between knowledge producers and users who provide information throughout the process. Involving people at the right time and place is essential to ensure the success of knowledge translation.*

Although knowledge translation interacts with a number of activities, including evidence-based health, continuing medical education, continuous professional development, and quality improvement, it is broader than all of them. It involves multiple factors present at different levels of the health system, which influence the way evidence is used by parties involved in decision-making^11^. Activities for knowledge translation may not be sequential and begin at any of the phases of the process, which will be listed below.

### Evidence Generation

This generation is represented by individual primary studies, i.e. first-hand reports of research results. In general, they are not ready to be transferred to practice yet, although they are essential to support future research. Randomized and observational controlled studies are examples of these first-generation studies.

### Evidence Synthesis

The synthesis consists of compiling the results of individual studies to determine what is known about the subject. In the health field, systematic reviews are very common, with or without meta-analyses^12^.

### Evidence Transfer

The transfer concerns the transmission of knowledge to the potential user. Communication is fundamental and cultural and linguistic boundaries interfere in the process. At least two aspects can be identified, the diffusion and dissemination of evidence. Diffusion refers to the distribution of information, usually by traditional means such as journal publications, conference presentation, and various activities based on the web (e.g., posts, blogs)^13^.

Dissemination extends beyond information by packaging the message to a specific audience^13^. It includes active and targeted methods such as art-based activities (e.g., development of video clips, dramaturgy) and use of knowledge disseminators (people who appropriate evidence and promote it within their own organization or in other environments)^14^.

### Evidence Implementation

The implementation focuses on strategies used to adopt and integrate evidence-based interventions and on how they work in certain scenarios. It emphasizes the importance of external validity (i.e. the degree to which the results of a study may be generalizable and relevant to populations other than those in which the original studies were conducted) and scalability (i.e. expansion of evidence-based practices to benefit more people and populations)^15,16^. Table 2 shows the main components of the implementation^17^.

**Table 2:** Main components of evidence implementation.

Steps	Description
To identify the problem	To critically analyze the literature on a problem, as well as identify knowledge gaps that justify practical implementation.
To adapt knowledge to the local context	To review the knowledge production, considering validity, usefulness and adaptation of results for a given situation, group or individual.
To assess barriers to the use of knowledge	To understand the difficulties in the assimilation and applicability of knowledge, as well as strategies to overcome such barriers.
To select, adapt and implement interventions	To plan and execute evidence-based interventions that promote knowledge implementation.
To monitor the use of knowledge	To assess whether knowledge was adequate for a given group, and whether there are new barriers to be considered.
To assess results	To determine the impact of the use of knowledge on health practices or the public system.
To support the use of knowledge	To plan the dissemination and expansion of knowledge. To assess whether the new knowledge continues to be used after the initial implementation.

## CHALLENGES AND PERSPECTIVES OF KNOWLEDGE TRANSLATION IN BRAZIL

The study of the theme in Brazil faces difficulties that are common to low- and middle-income countries. These difficulties are related to the local reality, characterized by low level of infrastructure and little engagement of people to translate evidence into practices, policies or programs^18^. There is also the restricted interaction between researchers and health decision-makers.

The health field has numerous impasses for the transfer and utilization of a new discovery. One of the major obstacles is the weakness of the research culture within the Brazilian Unified Health System (SUS). The interaction between knowledge producers and users should be increasingly stimulated from the identification of health problems, as it helps research agendas to be relevant to that context.

Some initiatives aim at identifying national and regional health needs and increase selective induction for relevant knowledge production – for example, the development of the National Agenda of Priorities in Health Research (NAPHR)^19^. It is relevant to systematize the process of defining health research priorities, in order to make it more transparent and stimulate the participation of public administrators, health professionals, politicians and the civil community in this construction^19^.

Brazil has secondary data collected in various forms by information systems and surveys^20^. These are valuable local evidence to assist in decision-making. However, the use of these data is limited due to the restricted skills of critical analysis and interpretation of evidence by health decision-makers. Furthermore, understanding what the information does not respond to is as important as what it can elucidate when analyzing a database. And since the amount of information missing is, most of the time, greater than the information available, it is necessary to ask the right questions. Disseminators of knowledge could play a role of providers of evidence and, thus, assist evidence-informed decision-making in the clinical area or in the management of services ^21^. Some argue that national funding agencies need to be more supportive of evaluative studies for dissemination and implementation of the knowledge produced. This way, the advancement of the practice in the public health field could be promoted^22^.

The lack of institutionalization in the use of evidence is a difficulty to be overcome. The World Health Organization has encouraged the use of evidence in health decision-making processes. One of the initiatives was the creation of the Evidence-Informed Policy Network (EVIPNet, https://www.who.int/evidence/en/). This initiative had repercussions in Brazil. EVIPNet Brasil prepares evidence syntheses for policies and deliberative dialogues to discuss the results of the syntheses^23^.

The evidence synthesis is part of the process of knowledge translation that assists the decision-making process, but it is not enough to ensure evidence-informed decision making alone^24^. Political and economic interests can hinder this process^25^, and the presence of institutional leaders who value the use of evidence facilitates its adoption^26^. In Brazil, the applicability of knowledge translation differs due to the peculiarity of the management of the system in three decision-making spheres. With municipalization, each local administrator manages a local health system with discretion, that is, administrators have freedom of choice, based on convenience and opportunity, to base or not their decision on evidence. Another challenge to be overcome concerns knowledge transfer, that is, the packaging of the main message in products that are easily assimilated by different audiences. In this sense, some actions were developed in Brazil, for example: clinical protocols and therapeutic guidelines (http://www.saude.gov.br/protocolos-e-diretrizes), the primary health care portal (https://aps.saude.gov.br/), the community of primary care practices (https://novo.atencaobasica.org.br/), and the Brazilian Cochrane Center (https://brazil.cochrane.org/). However, most of these actions are focused on diffusion.

It is expected to advance in strategies for dissemination of evidence that consider the cultural differences of Brazil. For example, evidence transfer in the Northeast can be performed by means of a *cordel* chapbook, depending on the issue involved. In addition, researchers should be encouraged to submit plans for knowledge translation as part of their grant proposals and improve the communication of their research results to the general public or establish partnerships with communication professionals and graphic designers^27^.

There are different strategies for knowledge translation, however, most were proposed and evaluated in developed countries^28^. Common characteristics for the success of these techniques include strong qualification, which considers the cultural, political and economic context and encourages a collaborative approach between researchers and decision-makers^28^.

It is easier to implement research after receiving qualification for the use of evidence^29^. A Brazilian initiative to sensitize and qualify administrators for the use of evidence is the specialization in evidence-informed policy (Espie), promoted by the Ministry of Health. This example could be expanded to benefit the whole country.

The implementation of evidence in the Brazilian public health is advancing slowly, mainly because there is still not enough skilled scientists and professionals. It is necessary to qualify epidemiologists for knowledge translation, in order to contribute effectively to the integration of evidence in practice, as they are among the main evidence generators. Epidemiology can boost implementation by providing evidence on effective interventions, as well as informing methods, impact indicators and design of implementation studies^30^.

## FINAL CONSIDERATIONS

Brazil began to move towards the process of applying scientific knowledge to real life. Investing in pilot studies to adapt knowledge translation interventions to the Brazilian scenario may be an alternative. Qualifying Brazilian researchers in the design and evaluation of implementation studies is relevant to improve this field in the country.
