# Supplementary material for: Knowledge translation in the reality of Brazilian public health
Source: Rev Saude Publica. 2020 Jul 20;54:72. doi: 10.11606/s1518-8787.2020054002073 (PMC7371410; doi:10.11606/s1518-8787.2020054002073)
Supplement: Translation

# Translation: Tradução do conhecimento na realidade da saúde pública brasileira

**Authors:** Keitty Regina Cordeiro de Andrade, Maurício Gomes Pereira

**Affiliations:** I Brasil Universidade de Brasília. Faculdade de Medicina. Departamento de Medicina. Brasília, DF, Brasil

**Keywords:** Ciência da Implementação, Disseminação de Informação, Pesquisa Médica Translacional, Comunicação em Saúde, Saúde Pública

## Abstract

O termo tradução do conhecimento tem sido utilizado para descrever o processo de aplicar os resultados de pesquisa no mundo real, com o intuito de potencializar a qualidade e a eficácia dos serviços e cuidados de saúde. O objetivo deste artigo é discutir a incorporação da tradução do conhecimento na saúde pública do Brasil. No artigo, aborda-se as atividades básicas da tradução do conhecimento e elenca-se desafios e perspectivas no cenário brasileiro. O Brasil começou a caminhar no entendimento do processo de traduzir o conhecimento científico para a prática. Investir em estudos pilotos para adaptar intervenções efetivas, assim rotuladas em outros países, para o cenário brasileiro pode ser uma alternativa. Aumentar a capacitação de pesquisadores brasileiros no delineamento e avaliação de estudos de implementação é relevante para a melhoria deste campo no País.

## INTRODUÇÃO

A aplicação de resultados de pesquisas na vida real é um desafio antigo que permanece no mundo contemporâneo^1^. Tradicionalmente, muitos anos decorrem para implementar um novo conhecimento, o que torna difícil a inovação na prestação de serviços de saúde, resulta em ineficiência dos sistemas de saúde e impacta na qualidade de vida da população^2^. Face a essa dificuldade, surgiram esforços para que as evidências sejam efetivamente compreendidas e implementadas nas práticas de saúde^3^.

Não há consenso entre os termos usados para descrever esses esforços^4^. Utilização da pesquisa (*research utilization*), ciência da implementação (*implementation science*), tradução do conhecimento (*knowledge translation*), transferência de conhecimento (*knowledge transfer*) e mobilização do conhecimento (*knowledge mobilization*) são expressões frequentemente empregadas^4-6^. Há ainda a sugestão de uso do K* (*knowledge star)*^7^. Neste artigo utilizaremos tradução do conhecimento por ser o mais utilizado mundialmente^8^.

Alguns países de média e baixa renda têm avançado na compreensão e na execução de estratégias para tradução do conhecimento^9^. E no Brasil? Quais os obstáculos e avanços da tradução do conhecimento? Utilizam-se resultados das pesquisas para formular diretrizes para práticas, políticas e programas? As diretrizes para a prática e as opções para políticas e programas são implementadas? Tais indagações motivaram a elaboração deste artigo, que tem o intuito de discutir conceitos que contribuem para descrever algumas das complexidades que influenciam a tradução do conhecimento para a saúde pública no Brasil.

## O QUE É A TRADUÇÃO DO CONHECIMENTO?

Tradução do conhecimento é um dos vários termos usados para descrever a ciência de colocar a evidência em ação e de entender como as práticas baseadas em evidências funcionam no mundo real. Trata-se de um processo interativo do conhecimento que inclui a síntese, a disseminação, o intercâmbio e a utilização do conhecimento com a finalidade de melhorar serviços e colocar à disposição da população produtos eficazes e, assim, fortalecer o sistema de saúde. A Tabela 1 apresenta os elementos que compõem uma definição de tradução do conhecimento e suas respectivas descrições^7,9^.

**Tabela 1:** Elementos que compõem a definição de tradução do conhecimento e a descrição de cada um.

Elementos do conhecimento	Descrição
Síntese	Contextualização e integração dos resultados de pesquisas individuais sobre o tema.
Disseminação	Transferência do conhecimento por meio da identificação do público-alvo, personalização da mensagem e definição dos melhores meios de comunicação.
Intercâmbio	Interação entre produtores e usuários do conhecimento, o com o intuito de aprendizagem mútua por uma parceria ativa para solucionar um determinado problema.
Aplicação	Uso do conhecimento na vida real por meio de atividades consistentes com princípios éticos e culturais, bem como estruturas legais e regulamentares.

Nota: Adaptada de Straus et al.^**5**^

Existem inúmeros modelos propostos para representar os componentes necessários para a tradução do conhecimento^10^. Os aspectos comuns entre eles se concentram na sugestão de ultrapassar os moldes tradicionais de divulgação da nova descoberta^11^. A Figura ilustra uma rotatória do conhecimento, análoga a uma rotatória de trânsito, onde o fluxo contínuo de tráfego ao redor da ilha central abrange fases dinâmicas para uso da evidência. Representa a ideia do conhecimento em movimento, que ao ser sintetizado e transferido é, presumidamente, melhor implementado pelos usuários.

**Figura:** Rotatória da tradução do conhecimento. O fluxo contínuo de tráfego ao redor da ilha central representa as atividades para a tradução do conhecimento que podem iniciar em qualquer fase do processo. São elas: *geração da evidência*, compreende os estudos primários imprescindíveis para apoiar as pesquisas futuras*; síntese da evidência*, compila os resultados de estudos primários para determinar o que é conhecido sobre um problema e interpretá-los no contexto da evidência global; *transferência da evidência*, emprega estratégias de difusão ou disseminação do conhecimento ao potencial usuário; *e implementação da evidência*, utiliza métodos para adotar intervenções baseadas em evidências, e entender como e por que elas funcionam em determinados contextos. Os veículos que entram e saem da rotatória representam a interação entre os produtores e usuários do conhecimento que fornecem informações ao longo do processo. Envolver as pessoas no momento e no lugar certo é essencial para garantir o sucesso da tradução do conhecimento.

Embora a tradução do conhecimento interaja com uma série de atividades, incluindo saúde baseada em evidências, educação médica continuada, desenvolvimento profissional contínuo e melhoria da qualidade, pode ser entendida como algo mais amplo que todas elas. Envolve múltiplos fatores presentes nos diferentes níveis do sistema de saúde, os quais influenciam a maneira como as evidências são usadas por partes envolvidas na tomada de decisão^11^. As atividades para tradução do conhecimento podem não ser sequenciais e se iniciar em qualquer uma das fases do processo, as quais serão listadas a seguir.

### Geração da Evidência

Essa geração é representada pelos estudos individuais primários, ou seja, os relatos em primeira mão dos resultados de pesquisa. Em geral, eles ainda não estão prontos para serem transferidos para a prática, embora sejam imprescindíveis para apoiar pesquisas futuras. Estudos randomizados e observacionais controlados são exemplos desses estudos de primeira geração.

### Síntese da Evidência

A síntese consiste em compilar os resultados de pesquisas individuais para determinar o que é conhecido sobre o tema. Na área da saúde, os tipos mais comuns são as revisões sistemáticas com ou sem metanálises ^12^.

### Transferência da Evidência

A transferência diz respeito à transmissão do conhecimento ao potencial usuário. A comunicação é fundamental e fronteiras culturais e linguísticas interferem no processo. Pelo menos dois aspectos podem ser identificados, a difusão e a disseminação da evidência. A difusão refere-se à distribuição da informação, geralmente por meios tradicionais como publicações em periódicos, apresentação em conferências e atividades diversas baseadas na *web* (por exemplo, postagens, blogs)^13^.

A disseminação amplia a comunicação da informação por empacotar a mensagem para um público-alvo específico^13^. Inclui métodos ativos e direcionados como atividades baseadas em arte (por exemplo, desenvolvimento de videoclipes, dramaturgia) e uso de disseminadores de conhecimento (pessoas que se apropriam das evidências e as promovem dentro de sua própria organização ou em outros ambientes)^14^.

### Implementação da Evidência

A implementação concentra-se em estratégias utilizadas para adotar e integrar intervenções baseadas em evidências, e entender como elas funcionam em determinados cenários. Enfatiza a importância da validade externa (ou seja, o grau em que os resultados de um estudo podem ser generalizáveis e relevantes para populações diferentes daqueles em que os estudos originais foram realizados) e da escalabilidade (ou seja, ampliação das práticas baseadas em evidências para beneficiar mais pessoas e populações)^15,16^. A Tabela 2 apresenta os principais componentes da implementação^17^.

**Tabela 2:** Principais componentes da implementação de evidências.

Etapas	Descrição
Identificar um problema	Analisar criticamente a literatura sobre um problema, bem como identificar lacunas no conhecimento que justifiquem a implementação prática.
Adaptar o conhecimento ao contexto local	Revisar a produção do conhecimento, levando em conta validade, utilidade e adaptação de resultados para determinada situação, grupo ou indivíduo.
Avaliar barreiras para o uso do conhecimento	Compreender as dificuldades à assimilação e à aplicabilidade do conhecimento, bem como estratégias para superar tais barreiras.
Selecionar, adaptar e implementar intervenções	Planejar e executar intervenções baseadas em evidências que promovam a implementação do conhecimento.
Monitorar o uso do conhecimento	Avaliar se o conhecimento foi adequado para determinado grupo, e ainda se há novas barreiras a serem consideradas.
Avaliar os resultados	Determinar o impacto do uso do conhecimento nas práticas em saúde ou no sistema público.
Sustentar o uso do conhecimento	Planejar a disseminação e a ampliação do uso do conhecimento. Avaliar se o novo conhecimento continua a ser usado após a implementação inicial.

## DESAFIOS E PERSPECTIVAS DA TRADUÇÃO DO CONHECIMENTO NO CENÁRIO BRASILEIRO

O estudo do tema no Brasil apresenta dificuldades que são comuns a países de baixa e média renda. Essas dificuldades se relacionam à realidade local, caracterizada por baixo nível de infraestrutura e pouco engajamento das pessoas para traduzir evidências em práticas, políticas ou programas^18^. Há também a restrita interação entre pesquisadores e tomadores de decisão em saúde.

Há numerosos impasses na área da saúde para a transferência e a utilização de uma nova descoberta. Um dos grandes obstáculos refere-se à debilidade da cultura de pesquisa no âmbito do Sistema Único de Saúde (SUS). A interação entre produtores e usuários do conhecimento deve ser cada vez mais estimulada a partir da identificação dos problemas de saúde, pois facilita que as agendas de pesquisa sejam relevantes para aquele contexto.

Existem iniciativas com intuito de identificar as necessidades nacionais e regionais de saúde e aumentar a indução seletiva para a produção relevante do conhecimento – por exemplo, o desenvolvimento da Agenda Nacional de Prioridades de Pesquisa em Saúde (ANPPS)^19^. É relevante sistematizar o processo de definição de prioridades de pesquisa em saúde, a fim de torná-lo mais transparente e de estimular a participação de gestores públicos, profissionais de saúde, políticos e comunidade civil nessa construção^19^.

O Brasil dispõe de dados secundários coletados de diversas formas por sistemas de informação e inquéritos ^20^. São evidências locais valiosas para auxiliar na tomada de decisão. No entanto, o uso desses dados é limitado devido às habilidades restritas de análise crítica e interpretação das evidências pelos decisores em saúde. Além disso, ao analisar uma base de dados, compreender o que as informações não respondem é tão importante quanto o que elas podem elucidar. E como a quantidade de informação faltante é, na maioria das vezes, maior do que a informação a disponível, é necessário fazer as perguntas certas. Disseminadores do conhecimento poderiam exercer um papel de provedores de evidências, e assim, auxiliar a tomada de decisão informada por evidências, na área clínica ou na gestão de serviços ^21^. Argumenta-se que as agências nacionais financiadoras de pesquisas necessitam avançar no apoio a estudos avaliativos para disseminação e implementação do conhecimento produzido. Dessa maneira, poderiam promover o avanço da sua prática no campo da saúde pública^22^.

A falta da institucionalização do uso de evidências é uma dificuldade a ser superada. A Organização Mundial de Saúde tem estimulado o uso de evidências em processos decisórios de saúde. Uma das iniciativas foi o a criação da Rede de Políticas Informadas por Evidências (do inglês *Evidence-Informed Policy Network– EVIPNet, https://www.who.int/evidence/en/*). Essa iniciativa repercutiu no Brasil. A EVIPNet Brasil elabora sínteses de evidências para políticas e diálogos deliberativos para discussão dos resultados das sínteses^23^.

Usar síntese de evidências é parte do processo de tradução do conhecimento que auxilia o processo decisório, mas não é suficiente por si só para garantir a tomada de decisão informada por evidências^24^. Interesses políticos e econômicos podem dificultar esse processo^25^, e a presença de lideranças institucionais que valorizem o uso de evidências facilitam a sua adoção^26^. No Brasil, a aplicabilidade da tradução do conhecimento se diferencia em função da peculiaridade da gestão do sistema em três esferas de decisão. Com a municipalização, cada gestor local administra um sistema local de saúde com poder discricionário, ou seja, o gestor tem liberdade de escolha, pautada na conveniência e oportunidade, para basear ou não sua decisão em evidências. Outro desafio a ser superado está na transferência do conhecimento, ou seja, no empacotamento da mensagem principal em produtos que sejam facilmente assimilados por diferentes públicos. Nesse sentido, algumas ações foram desenvolvidas no cenário brasileiro, por exemplo: os protocolos clínicos e diretrizes terapêuticas (http://www.saude.gov.br/protocolos-e-diretrizes), o portal da atenção primária à saúde (https://aps.saude.gov.br/), a comunidade de práticas da atenção básica (https://novo.atencaobasica.org.br/) e o Centro Cochrane Brasil (https://brazil.cochrane.org/). Contudo, a maioria dessas ações é voltada para a difusão.

Espera-se o avanço em estratégias de disseminação de evidências que considerem as diferenças culturais do Brasil. Por exemplo, a transferência de evidências no Nordeste pode ser realizada por meio de um cordel, a depender da questão envolvida. Além disso, os pesquisadores devem ser encorajados a apresentar planos para tradução do conhecimento como parte de suas propostas de subsídios e aperfeiçoar a comunicação dos resultados de suas pesquisas para o público em geral ou estabelecer parcerias com profissionais de comunicação e designers gráficos^27^.

Existem diferentes estratégias para tradução do conhecimento, no entanto, a maioria foi proposta e avaliada em países desenvolvidos^28^. As características comuns para o sucesso dessas técnicas incluem um forte treinamento ou elemento de capacitação, que considere o contexto cultural, político e econômico e encoraje uma abordagem colaborativa entre pesquisadores e tomadores de decisão^28^.

Há maior facilidade para implementar pesquisas quando se recebe treinamento sobre uso de evidências^29^. Uma iniciativa brasileira para sensibilizar e capacitar gestores sobre o uso de evidências é a especialização em política informada por evidências (Espie), promovido pelo Ministério da Saúde. Esse exemplo poderia ser expandido de modo a beneficiar todo o País.

A implementação de evidências em saúde pública no Brasil está avançando lentamente, principalmente porque ainda não existe um quadro suficiente de cientistas e profissionais com a capacidade apropriada. Faz-se necessário aumentar a capacitação dos epidemiologistas sobre tradução do conhecimento, a fim de contribuir efetivamente para a integração das evidências na prática, pois estão entre os principais geradores de evidências. A epidemiologia pode impulsionar a implementação ao fornecer evidências sobre intervenções efetivas, bem como informar métodos, indicadores de impacto e delineamentos dos estudos de implementação^30^.

## CONSIDERAÇÕES FINAIS

O Brasil começou a caminhar no processo de aplicar o conhecimento científico na vida real. Investir em estudos pilotos para adaptar ao cenário brasileiro intervenções de tradução do conhecimento efetivas em outros países pode ser uma alternativa. Aumentar a capacitação de pesquisadores brasileiros no delineamento e avaliação de estudos de implementação é relevante para a melhoria deste campo no País.
